# 3-Methyl-5-(4-methyl­phen­oxy)-1-phenyl-1*H*-pyrazole-4-carbaldehyde

**DOI:** 10.1107/S2414314622009245

**Published:** 2022-09-27

**Authors:** Sreeramapura D. Archana, Holalagudu A. Nagma Banu, Balakrishna Kalluraya, Hemmige S. Yathirajan, Rishik Balerao, Ray J. Butcher

**Affiliations:** a Department of Studies in Chemistry University of Mysore, Manasagangotri, Mysore-570 006, India; b Department of Studies in Chemistry Mangalore University Mangalagangotri, Mangalore-574 199, India; c Thomas Jefferson High School for Science and Technology, 6560 Braddock Rd Alexandria VA 22312, USA; d Department of Chemistry, Howard University, 525 College Street NW, Washington DC 20059, USA; University of Aberdeen, Scotland

**Keywords:** crystal structure, pyrazole, phen­yl, aldehyde

## Abstract

In the title compound, C_18_H_16_N_2_O_2_, the phenyl and pyrazole rings subtend a dihedral angle of 22.68 (8)°. The packing of the title compound features aromatic π–π stacking and weak C—H⋯π inter­actions.

## Abstract 2

In the title compound, the phenyl and pyrazole rings subtend a dihedral angle of 22.68 (8)°.

## Graphical abstract

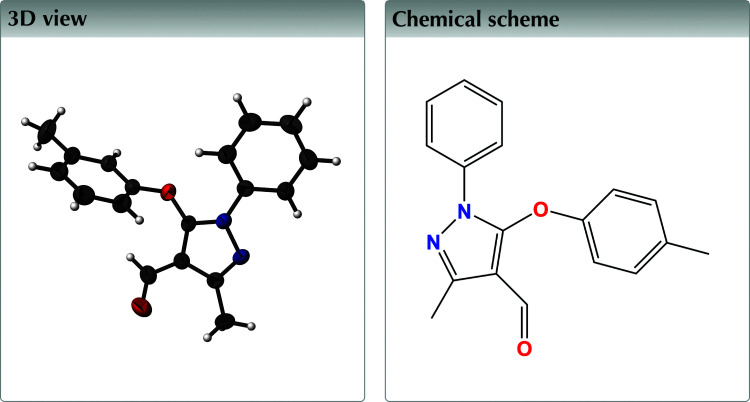

## Structure description

Pyrazoles possess many pharmacological activities such as the inhibition of protein glycation, anti­bacterial, anti­fungal, anti­cancer, anti­depressant, anti-inflammatory, anti-tuberculosis and anti­oxidant activity as well as being used as anti­viral agents (Fustero *et al.*, 2011 ▸; Steinbach *et al.*, 2000 ▸; García-Lozano *et al.*, 1997 ▸). The crystal structures of (*E*)-1,3-dimethyl-5-*p*-tol­yloxy-1*H*-pyrazole-4-carbaldehyde *o*-(6-chloro­pyridazin-3-yl)oxime (Hu *et al.*, 2006 ▸), 1-(5-bromo­pyrimidin-2-yl)-3-phenyl-1*H*-pyrazole-4-carbaldehyde (Thiruvalluvar *et al.*, 2007 ▸), 5-(2,4-di­chloro­phen­oxy)-3-methyl-1-phenyl-1*H*-pyrazole-4-carbaldehyde (Kumar *et al.*, 2016 ▸), four 1-aryl-1*H*-pyrazole-3,4-di­carbox­ylate derivatives (Asma *et al.*, 2018 ▸), functionalized 3-(5-ar­yloxy-3-methyl-1-phenyl-1*H*-pyrazol-4-yl)-1-(4-substituted-phen­yl)prop-2-en-1-ones (Kiran Kumar *et al.*, 2020 ▸) and two isostructural 3-(5-ar­yloxy-3-methyl-1-phenyl-1*H*-pyrazol-4-yl)-1-(thio­phen-2-yl)prop-2-en-1-ones (Shaibah *et al.*, 2020 ▸) have been reported.

**Figure 1:**
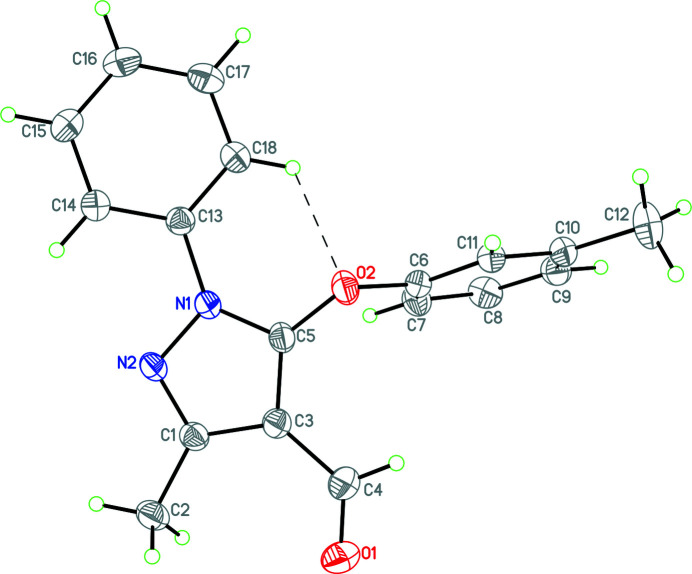
Diagram of **1** showing displacement ellipsoids at the 30% probability level. The C18—H18⋯O2 intra­molecular contact is shown by a dashed line.

As part our studies in this area, we now report the synthesis and crystal structure of the title compound, C_18_H_16_N_2_O_2_, (**1**, Fig. 1 ▸) . Compound **1** crystallizes in the monoclinic space group *P*2_1_/*c* with one mol­ecule in the asymmetric unit. It consists of a C1/C3/C5/N1/N2 pyrazole ring linked to a C13–C18 phenyl ring by a carbon–nitro­gen single bond [C13—N1 = 1.4285 (17) Å]. As a result of the single bond, the pyrazol and phenyl rings are twisted with a dihedral angle of 22.68 (8)°, perhaps due to the steric inter­action between H18 and O2 and between H14 and N2. In the pyrazole ring, the aldehyde group (C3/C4/O1) is slightly twisted with a dihedral angle of 6.43 (10)° as a result of the steric inter­action of this group with the C2 methyl substituent. The C6–C12 toluyl substituent makes dihedral angles of 79.44 (5) and 82.40 (5)° with the pyrazol and phenyl rings, respectively. A short intra­molecular C18—H18⋯O2 contact closes an *S*(6) ring.

**Figure 2:**
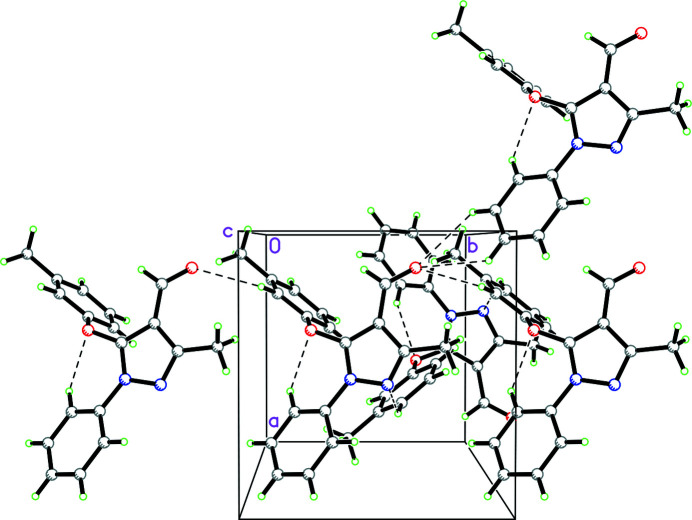
Packing diagram for **1** (viewed along the *c*-axis direction) showing C—H⋯O and C—H⋯N contacts as dashed lines.

In the packing, a very weak C11—H11⋯O1 hydrogen bond generates [010] chains (Fig. 2 ▸, Table 1 ▸). In addition, aromatic π–π stacking involving the pyrazole rings in adjacent mol­ecules [centroid-to-centroid distance = 3.8908 (9) Å, slippage = 1.233 Å, symmetry operation 1 − *x*, 1 − *y*, 1 − *z*] is observed. There is also a weak C—H⋯π inter­action involving the toluene rings in adjacent mol­ecules [distance between ring centroid and carbon atom = 3.8075 (17) Å, C—H⋯*Cg* = 148°, symmetry operation 1 − *x*, 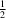 + *y*, 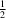 − *z*].

## Synthesis and crystallization

**Figure 3:**
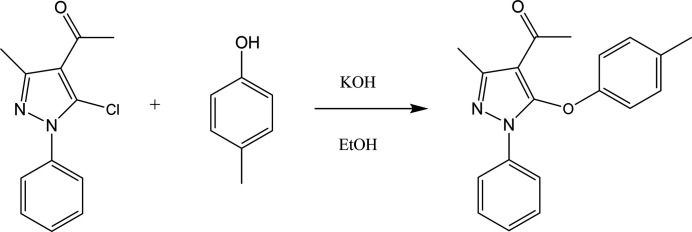
Reaction scheme.

To a solution of *p*-cresol (0.1 mol) dissolved in 10 ml of di­methyl­sulfoxide,1-phenyl-5-chloro-3-methyl-1*H*-pyrazol-4-carbaldehyde (3.22 g, 0.1 mol) and potassium hydroxide (0.8 g, 0.1 mol) were added and the resulting solution was heated on a water bath for 5 h. The reaction mixture was cooled to room temperature and poured onto crushed ice. The solid that separated was filtered off and washed with water and the dried product was recrystallized from ethanol solution. The reaction scheme is shown in Fig. 3 ▸.

Yield: 82%; m.p. 320–322 K; MS (*m*/*z*) 293.1 (*M* ^+^ + 1). ^1^H NMR (400 MHz, CDCl_3_, δ p.p.m.), 2.22 (*s*, 3H, pyrazole meth­yl), 2.34 (*s*, 3H, *o*-tol­yloxy meth­yl), 6.93 (*d*, 2H, *J* = 8.3 Hz, Ar—H), 7.04 (*d*, 2H, *J* = 8.3 Hz, Ar—H), 7.23 (*d*, 1H, *J* = 7.3 Hz), 7.46 (*d*, 2H, *J* = 8.1 Hz, Ar—H), 7.81 (*d*, 2H, *J* = 8.1 Hz, Ar—H), 8.61 (*s*, 1H, aldehyde-H).

## Refinement

Crystal data, data collection and structure refinement details are summarized in Table 2 ▸.

## Supplementary Material

Crystal structure: contains datablock(s) I. DOI: 10.1107/S2414314622009245/hb4413sup1.cif

Structure factors: contains datablock(s) I. DOI: 10.1107/S2414314622009245/hb4413Isup2.hkl

Supporting information file. DOI: 10.1107/S2414314622009245/hb4413Isup3.cml

CCDC reference: 2207948

Additional supporting information:  crystallographic information; 3D view; checkCIF report

## Figures and Tables

**Table 1:** Hydrogen-bond geometry (Å, °)

*D*—H⋯*A*	*D*—H	H⋯*A*	*D*⋯*A*	*D*—H⋯*A*
C18—H18⋯O2	0.95	2.42	2.9593 (19)	116
C11—H11⋯O1^i^	0.95	2.75	3.626 (2)	153

Symmetry code: (i) 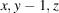 .

**Table 2:** Experimental details

Crystal data	
Chemical formula	C_18_H_16_N_2_O_2_
*M*_r_	292.33
Crystal system, space group	Monoclinic, *P*2_1_/*c*
Temperature (K)	100
*a*, *b*, *c* (Å)	8.2745 (5), 7.9167 (6), 23.0663 (17)
β (°)	93.225 (4)
*V* (Å^3^)	1508.60 (18)
*Z*	4
Radiation type	Mo *K*α
μ (mm^−1^)	0.09
Crystal size (mm)	0.33 × 0.29 × 0.21

Data collection	
Diffractometer	Bruker APEXII CCD
Absorption correction	Multi-scan (*SADABS*; Krause *et al.*, 2015 ▸)
*T*_min_, *T*_max_	0.560, 0.746
No. of measured, independent and observed [*I* > 2σ(*I*)] reflections	22729, 4612, 3061
*R*_int_	0.075
(sin θ/λ)_max_ (Å^−1^)	0.717

Refinement	
*R*[*F* ^2^ > 2σ(*F* ^2^)], *wR*(*F* ^2^), *S*	0.051, 0.150, 1.08
No. of reflections	4612
No. of parameters	201
H-atom treatment	H-atom parameters constrained
Δρ_max_, Δρ_min_ (e Å^−3^)	0.24, −0.22

Computer programs: *APEX2* (Bruker, 2005 ▸), *SAINT* (Bruker, 2002 ▸), *SHELXT* (Sheldrick 2015*a* ▸), *SHELXL2018/3* (Sheldrick, 2015*b* ▸) and *OLEX2* (Dolomanov *et al.*, 2009 ▸).

## full crystallographic data

### Crystal data

**Table:**

C_18_H_16_N_2_O_2_	*F*(000) = 616
*M**_r_* = 292.33	*D*_x_ = 1.287 Mg m^−^^3^
Monoclinic, *P*2_1_/*c*	Mo *K*α radiation, λ = 0.71073 Å
*a* = 8.2745 (5) Å	Cell parameters from 6530 reflections
*b* = 7.9167 (6) Å	θ = 3.1–29.6°
*c* = 23.0663 (17) Å	µ = 0.09 mm^−^^1^
β = 93.225 (4)°	*T* = 100 K
*V* = 1508.60 (18) Å^3^	Block, pale yellow
*Z* = 4	0.33 × 0.29 × 0.21 mm

### Data collection

**Table:**

Bruker APEXII CCD diffractometer	3061 reflections with *I* > 2σ(*I*)
φ and ω scans	*R*_int_ = 0.075
Absorption correction: multi-scan (SADABS; Krause *et al.*, 2015)	θ_max_ = 30.6°, θ_min_ = 2.5°
*T*_min_ = 0.560, *T*_max_ = 0.746	*h* = −11→11
22729 measured reflections	*k* = −11→9
4612 independent reflections	*l* = −32→32

### Refinement

**Table:**

Refinement on *F*^2^	0 restraints
Least-squares matrix: full	Hydrogen site location: inferred from neighbouring sites
*R*[*F*^2^ > 2σ(*F*^2^)] = 0.051	H-atom parameters constrained
*wR*(*F*^2^) = 0.150	*w* = 1/[σ^2^(*F*_o_^2^) + (0.0554*P*)^2^ + 0.2283*P*] where *P* = (*F*_o_^2^ + 2*F*_c_^2^)/3
*S* = 1.08	(Δ/σ)_max_ < 0.001
4612 reflections	Δρ_max_ = 0.24 e Å^−^^3^
201 parameters	Δρ_min_ = −0.21 e Å^−^^3^

### Special details

**Table:**

Geometry. All esds (except the esd in the dihedral angle between two l.s. planes) are estimated using the full covariance matrix. The cell esds are taken into account individually in the estimation of esds in distances, angles and torsion angles; correlations between esds in cell parameters are only used when they are defined by crystal symmetry. An approximate (isotropic) treatment of cell esds is used for estimating esds involving l.s. planes.
Refinement. All hydrogen atoms were placed geometrically and refined as riding atoms with their *U*_iso_ values 1.2 × (1.5 × for CH_3_) that of their attached atoms.

### Fractional atomic coordinates and isotropic or equivalent isotropic displacement parameters (Å^2^)

**Table:**

	*x*	*y*	*z*	*U*_iso_*/*U*_eq_	
O1	0.14639 (15)	0.70581 (16)	0.38374 (6)	0.0644 (3)	
O2	0.41488 (13)	0.23801 (12)	0.37711 (4)	0.0434 (3)	
N1	0.61932 (13)	0.41501 (13)	0.41767 (5)	0.0370 (3)	
N2	0.63851 (14)	0.57702 (14)	0.43941 (5)	0.0395 (3)	
C1	0.49777 (17)	0.65200 (17)	0.42911 (6)	0.0388 (3)	
C2	0.4761 (2)	0.83049 (19)	0.44777 (7)	0.0502 (4)	
H2A	0.576401	0.870910	0.467780	0.075*	
H2B	0.387621	0.836723	0.474185	0.075*	
H2C	0.450214	0.901154	0.413627	0.075*	
C3	0.38270 (17)	0.54160 (18)	0.40121 (6)	0.0398 (3)	
C4	0.21536 (19)	0.5708 (2)	0.38257 (7)	0.0492 (4)	
H4	0.154397	0.476467	0.368230	0.059*	
C5	0.46660 (16)	0.39239 (16)	0.39555 (6)	0.0373 (3)	
C6	0.36301 (16)	0.21805 (16)	0.31822 (6)	0.0358 (3)	
C7	0.41496 (19)	0.32023 (19)	0.27473 (7)	0.0453 (3)	
H7	0.486871	0.411651	0.283142	0.054*	
C8	0.3587 (2)	0.2851 (2)	0.21804 (7)	0.0510 (4)	
H8	0.393472	0.353203	0.187199	0.061*	
C9	0.25337 (19)	0.1535 (2)	0.20582 (6)	0.0473 (4)	
H9	0.214581	0.133416	0.166890	0.057*	
C10	0.20347 (17)	0.05002 (18)	0.25005 (6)	0.0424 (3)	
C11	0.26116 (15)	0.08271 (17)	0.30678 (6)	0.0372 (3)	
H11	0.230471	0.011963	0.337554	0.045*	
C12	0.0879 (2)	−0.0940 (3)	0.23791 (8)	0.0687 (5)	
H12A	0.086719	−0.123627	0.196639	0.103*	
H12B	−0.021126	−0.060187	0.247824	0.103*	
H12C	0.122673	−0.192035	0.261368	0.103*	
C13	0.75428 (16)	0.30235 (16)	0.42240 (6)	0.0371 (3)	
C14	0.87596 (18)	0.3355 (2)	0.46428 (7)	0.0470 (3)	
H14	0.867780	0.429849	0.489387	0.056*	
C15	1.0098 (2)	0.2308 (2)	0.46956 (8)	0.0554 (4)	
H15	1.093906	0.254084	0.498186	0.067*	
C16	1.0220 (2)	0.0931 (2)	0.43365 (8)	0.0540 (4)	
H16	1.113977	0.021373	0.437420	0.065*	
C17	0.8997 (2)	0.0602 (2)	0.39220 (8)	0.0566 (4)	
H17	0.907241	−0.035666	0.367730	0.068*	
C18	0.7662 (2)	0.1650 (2)	0.38573 (7)	0.0505 (4)	
H18	0.683509	0.143019	0.356447	0.061*	

### Atomic displacement parameters (Å^2^)

**Table:**

	*U*^11^	*U*^22^	*U*^33^	*U*^12^	*U*^13^	*U*^23^
O1	0.0580 (7)	0.0630 (8)	0.0711 (8)	0.0205 (6)	−0.0064 (6)	−0.0004 (6)
O2	0.0538 (6)	0.0350 (5)	0.0400 (5)	−0.0067 (4)	−0.0104 (4)	−0.0001 (4)
N1	0.0410 (6)	0.0327 (5)	0.0367 (6)	0.0002 (4)	−0.0036 (4)	−0.0047 (4)
N2	0.0464 (6)	0.0325 (5)	0.0391 (6)	−0.0002 (5)	−0.0018 (5)	−0.0069 (5)
C1	0.0459 (7)	0.0362 (7)	0.0343 (7)	0.0031 (6)	0.0017 (5)	−0.0008 (5)
C2	0.0614 (9)	0.0379 (7)	0.0512 (9)	0.0061 (7)	0.0014 (7)	−0.0067 (6)
C3	0.0430 (7)	0.0388 (7)	0.0371 (7)	0.0020 (6)	−0.0023 (5)	−0.0002 (5)
C4	0.0478 (8)	0.0526 (9)	0.0463 (8)	0.0051 (7)	−0.0060 (6)	0.0003 (7)
C5	0.0431 (7)	0.0343 (6)	0.0337 (6)	−0.0028 (5)	−0.0051 (5)	−0.0013 (5)
C6	0.0355 (6)	0.0352 (6)	0.0361 (6)	0.0020 (5)	−0.0026 (5)	−0.0026 (5)
C7	0.0492 (8)	0.0409 (7)	0.0458 (8)	−0.0069 (6)	0.0023 (6)	0.0007 (6)
C8	0.0635 (10)	0.0480 (8)	0.0422 (8)	0.0023 (7)	0.0080 (7)	0.0061 (7)
C9	0.0554 (9)	0.0496 (8)	0.0364 (7)	0.0078 (7)	−0.0025 (6)	−0.0052 (6)
C10	0.0400 (7)	0.0435 (7)	0.0431 (7)	0.0011 (6)	−0.0022 (5)	−0.0084 (6)
C11	0.0362 (6)	0.0374 (7)	0.0378 (7)	−0.0011 (5)	0.0006 (5)	−0.0018 (5)
C12	0.0738 (12)	0.0718 (12)	0.0589 (11)	−0.0261 (10)	−0.0100 (9)	−0.0122 (9)
C13	0.0395 (7)	0.0351 (6)	0.0366 (7)	0.0014 (5)	0.0020 (5)	0.0008 (5)
C14	0.0492 (8)	0.0456 (8)	0.0453 (8)	0.0040 (6)	−0.0061 (6)	−0.0054 (6)
C15	0.0495 (9)	0.0597 (10)	0.0557 (9)	0.0102 (7)	−0.0088 (7)	−0.0006 (8)
C16	0.0510 (9)	0.0535 (9)	0.0581 (10)	0.0158 (7)	0.0073 (7)	0.0061 (7)
C17	0.0617 (10)	0.0485 (9)	0.0601 (10)	0.0101 (7)	0.0085 (8)	−0.0112 (7)
C18	0.0515 (9)	0.0487 (8)	0.0507 (9)	0.0049 (7)	−0.0030 (7)	−0.0125 (7)

### Geometric parameters (Å, º)

**Table:**

O1—C4	1.2129 (19)	C8—C9	1.377 (2)
O2—C5	1.3555 (15)	C9—H9	0.9500
O2—C6	1.4103 (16)	C9—C10	1.389 (2)
N1—N2	1.3830 (15)	C10—C11	1.3917 (19)
N1—C5	1.3478 (17)	C10—C12	1.505 (2)
N1—C13	1.4285 (17)	C11—H11	0.9500
N2—C1	1.3167 (18)	C12—H12A	0.9800
C1—C2	1.4909 (19)	C12—H12B	0.9800
C1—C3	1.4197 (19)	C12—H12C	0.9800
C2—H2A	0.9800	C13—C14	1.381 (2)
C2—H2B	0.9800	C13—C18	1.384 (2)
C2—H2C	0.9800	C14—H14	0.9500
C3—C4	1.445 (2)	C14—C15	1.383 (2)
C3—C5	1.3800 (19)	C15—H15	0.9500
C4—H4	0.9500	C15—C16	1.376 (2)
C6—C7	1.376 (2)	C16—H16	0.9500
C6—C11	1.3794 (18)	C16—C17	1.377 (2)
C7—H7	0.9500	C17—H17	0.9500
C7—C8	1.391 (2)	C17—C18	1.383 (2)
C8—H8	0.9500	C18—H18	0.9500
			
C5—O2—C6	118.48 (10)	C8—C9—C10	120.45 (14)
N2—N1—C13	118.56 (11)	C10—C9—H9	119.8
C5—N1—N2	110.27 (11)	C9—C10—C11	118.70 (13)
C5—N1—C13	131.16 (11)	C9—C10—C12	121.51 (14)
C1—N2—N1	105.68 (11)	C11—C10—C12	119.79 (14)
N2—C1—C2	119.63 (13)	C6—C11—C10	119.87 (13)
N2—C1—C3	111.60 (12)	C6—C11—H11	120.1
C3—C1—C2	128.76 (13)	C10—C11—H11	120.1
C1—C2—H2A	109.5	C10—C12—H12A	109.5
C1—C2—H2B	109.5	C10—C12—H12B	109.5
C1—C2—H2C	109.5	C10—C12—H12C	109.5
H2A—C2—H2B	109.5	H12A—C12—H12B	109.5
H2A—C2—H2C	109.5	H12A—C12—H12C	109.5
H2B—C2—H2C	109.5	H12B—C12—H12C	109.5
C1—C3—C4	130.04 (13)	C14—C13—N1	118.10 (12)
C5—C3—C1	103.98 (12)	C14—C13—C18	120.17 (13)
C5—C3—C4	125.97 (13)	C18—C13—N1	121.72 (13)
O1—C4—C3	125.34 (15)	C13—C14—H14	120.1
O1—C4—H4	117.3	C13—C14—C15	119.77 (14)
C3—C4—H4	117.3	C15—C14—H14	120.1
O2—C5—C3	130.53 (13)	C14—C15—H15	119.8
N1—C5—O2	120.75 (12)	C16—C15—C14	120.46 (15)
N1—C5—C3	108.45 (11)	C16—C15—H15	119.8
C7—C6—O2	123.07 (12)	C15—C16—H16	120.3
C7—C6—C11	121.98 (13)	C15—C16—C17	119.48 (15)
C11—C6—O2	114.90 (12)	C17—C16—H16	120.3
C6—C7—H7	121.1	C16—C17—H17	119.6
C6—C7—C8	117.77 (14)	C16—C17—C18	120.83 (15)
C8—C7—H7	121.1	C18—C17—H17	119.6
C7—C8—H8	119.4	C13—C18—H18	120.4
C9—C8—C7	121.20 (14)	C17—C18—C13	119.28 (15)
C9—C8—H8	119.4	C17—C18—H18	120.4
C8—C9—H9	119.8		
			
O2—C6—C7—C8	178.25 (13)	C5—N1—C13—C14	−157.00 (15)
O2—C6—C11—C10	−179.47 (12)	C5—N1—C13—C18	24.1 (2)
N1—N2—C1—C2	179.50 (12)	C5—C3—C4—O1	−174.07 (16)
N1—N2—C1—C3	0.71 (15)	C6—O2—C5—N1	−118.79 (14)
N1—C13—C14—C15	−178.97 (14)	C6—O2—C5—C3	67.98 (19)
N1—C13—C18—C17	179.89 (14)	C6—C7—C8—C9	0.5 (2)
N2—N1—C5—O2	−173.26 (12)	C7—C6—C11—C10	−2.3 (2)
N2—N1—C5—C3	1.32 (16)	C7—C8—C9—C10	−1.4 (2)
N2—N1—C13—C14	21.84 (19)	C8—C9—C10—C11	0.4 (2)
N2—N1—C13—C18	−157.01 (13)	C8—C9—C10—C12	179.67 (16)
N2—C1—C3—C4	179.55 (14)	C9—C10—C11—C6	1.4 (2)
N2—C1—C3—C5	0.05 (16)	C11—C6—C7—C8	1.3 (2)
C1—C3—C4—O1	6.5 (3)	C12—C10—C11—C6	−177.89 (15)
C1—C3—C5—O2	173.04 (14)	C13—N1—N2—C1	179.67 (11)
C1—C3—C5—N1	−0.83 (15)	C13—N1—C5—O2	5.7 (2)
C2—C1—C3—C4	0.9 (3)	C13—N1—C5—C3	−179.76 (13)
C2—C1—C3—C5	−178.59 (14)	C13—C14—C15—C16	−0.5 (3)
C4—C3—C5—O2	−6.5 (2)	C14—C13—C18—C17	1.1 (2)
C4—C3—C5—N1	179.64 (14)	C14—C15—C16—C17	0.1 (3)
C5—O2—C6—C7	25.02 (19)	C15—C16—C17—C18	0.9 (3)
C5—O2—C6—C11	−157.83 (12)	C16—C17—C18—C13	−1.5 (3)
C5—N1—N2—C1	−1.26 (15)	C18—C13—C14—C15	−0.1 (2)

### Hydrogen-bond geometry (Å, º)

**Table:**

*D*—H···*A*	*D*—H	H···*A*	*D*···*A*	*D*—H···*A*
C18—H18···O2	0.95	2.42	2.9593 (19)	116
C11—H11···O1^i^	0.95	2.75	3.626 (2)	153

Symmetry code: (i) *x*, *y*−1, *z*.
